# Arthroscopic Linear Chain Fixation for the Treatment of Medial Meniscus Posterior Root Tear: A Case Study

**DOI:** 10.1111/os.13975

**Published:** 2024-01-05

**Authors:** Jing‐qing Chen, Hai‐yun Niu, Ming Li, Zhen‐yue Dong, Ju‐yuan Gu, Wei‐xia Bai, Bai‐cheng Chen

**Affiliations:** ^1^ Orthopedics Department of Joint Surgury the Third Hospital of Hebei Medical University Shijiazhuang China

**Keywords:** Arthroscopy, Medial meniscus, Meniscus posterior root tear, Repair surgery

## Abstract

**Background:**

The repair and reconstruction of medial meniscus posterior root tears (MMPRTs) is an important issue in the field of orthopedic sports medicine. This study reports the first application of arthroscopic linear chain fixation for the treatment of MMPRTs.

**Case presentation:**

A 78‐year‐old female patient presented with a 1.5‐month history of right knee pain accompanied by a locked facet joint. The patient underwent surgery with the new linear chain fixation method. In this method, the suture and the loop part of the buckle‐strap titanium plate were combined into a linear chain mechanical complex, and the tension of the posterior root stump was gradually increased by pulling on the two attachment lines at the external mouth of the tibial tunnel. The postoperative Lysholm score was 89, and the visual analogue scale score was 0.9, indicating a significant improvement in knee joint function. At the 7‐month and 1‐year post‐surgery follow‐up, physical and MRI examinations confirmed satisfactory healing of the MMPRTs.

**Conclusion:**

This surgical approach offers several benefits, including a simplified instrumentation setup, preservation of natural anatomical structures, and reliable residual stump fixation. It has the potential for clinical implementation.

## Graphical abstract

This graphical abstract showed the treatment and observation for the repair surgery of medial meniscus posterior root injuries. And this case report of arthroscopic linear cyclic fixation proposed good feasibility and clinical efficacy. And It has the potential for being applied clinically.

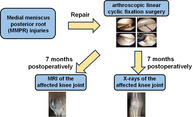

## Introduction

The meniscus serves to protect articular cartilage by increasing the contact area between the femoral condyles and the proximal tibial joint surface, thus reducing load and stress distribution on the joint surface. It also plays a significant role in lubrication.^1^ Meniscal tissue from patients with traumatic meniscal injury demonstrates similar extracellular matrix structures and pathological alterations characteristic of tissue from patients with late‐stage osteoarthritis, suggesting that meniscal tears can initiate and progress knee osteoarthritis.^2^, ^3^ Additionally, meniscus tears are often associated with inflammation of the synovial membrane, cartilage lesions and knee symptoms and pain.^4^, ^5^, ^6^

The posterior root of the medial meniscus is tightly connected to the tibial spine and hence, it has lower mobility than the lateral meniscus, making it more susceptible to traumatic injuries and degenerative changes than the lateral meniscus.^7^ Medial meniscus posterior root (MMPR) injuries are commonly seen in clinical sports injuries, with incidence rates accounting for 10%–36% of all meniscus injuries.^8^

Surgical reconstruction of the posterior root can enhance knee joint stability, decrease the peak pressure on the articular surface and slow the progression of osteoarthritis.^9^, ^10^ While partial meniscectomy was once considered the gold standard for treatment, meniscus root repair has become increasingly favored, with reported improvements in clinical and biomechanical outcomes. Two primary repair techniques have been described: suture anchors (direct fixation) and sutures inserted through a tibial tunnel (indirect fixation).^11^ However, intraoperative damage to articular cartilage and postoperative poor tension of the medial meniscus stump are crucial factors that lead to unsatisfactory surgical outcomes.^12^

In this study, combined with the authors' clinical practice, a new linear chain fixation method for the traditional transtibial fixation of MMPR repair has been developed. This method is presented below.

## Case Presentation

### Patient

**FIGURE 1:**
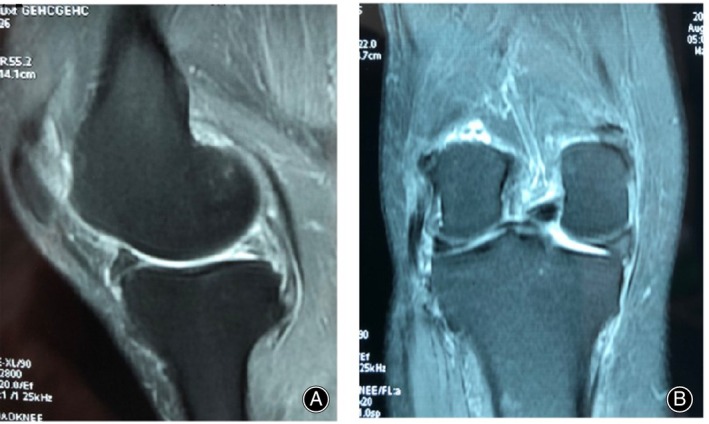
Preoperative magnetic resonance imaging (MRI) of the affected knee joint. (A) sagittal scans; (B) coronal scans.

A 78‐year‐old female patient presented with a 1.5‐month history of right knee pain accompanied by a locked facet joint. The patient had a 15‐year history of hypertension and a 12‐year history of diabetes. Physical examination: ambulation was impaired. There was slight swelling of the left knee, with a non‐weight‐bearing flexion–extension range of motion in the left knee of 0°–120°. Deep squatting was markedly limited after loading the left knee. Overextension and overflexion tests were positive, and tenderness in the medial and lateral compartments was also positive, as was McMurray's sign. The Lysholm score was 22 points, and the visual analogue scale (VAS) pain score was 5 points. Magnetic resonance imaging (MRI) of the affected knee showed a hollow‐liked deficit (Figure 1A) and a protrusion deformity (Figure 1B) in the posterior root of the medial meniscus, with osteoarthritis.

### Surgical Procedure

**FIGURE 2:**
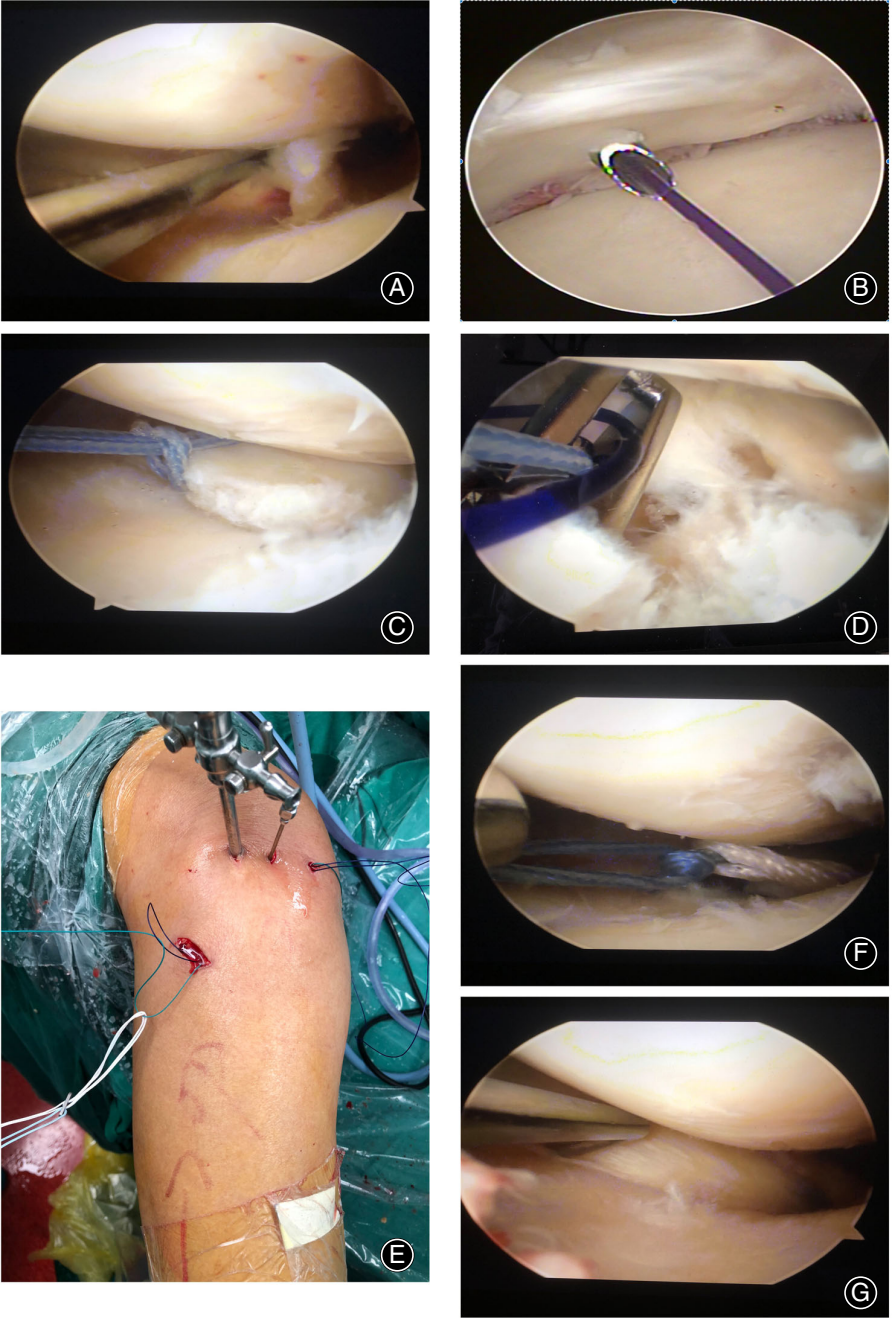
The process of repairing the endpoint of medial meniscus posterior root tears (MMPRT using arthroscopic linear chain fixation. (A) Arthroscopic exploration confirmed a complete rupture of the posterior root of the medial meniscus; (B) a high‐positioned approach from the posteromedial aspect of the knee was used to pass a cannulated needle through the MMRPT stump; (C) the medial meniscus stump was fixed using Ethibond surgical suture; (D) one looped end of the Ethibond surgical suture and one folded end of the polydioxanone (PDS) surgical suture were led out of the joint cavity to the external opening of the tibial tunnel; (E) after passing the Ethibond suture through the loop portion of the mini titanium plate at the external opening of the tunnel, it was prepared for combination with the folded end of the PDS suture; (F) by traction through the PDS suture, one end of the Ethibond suture was slipped into the joint cavity and then tied with the other end, together with the loop of the mini titanium plate forming a linear tension complex; and (G) tightening the adjustable loop secured the mini titanium plate at the external opening of the tibial tunnel. The stability and tension of the medial meniscus were satisfactory upon examination.

**FIGURE 3:**
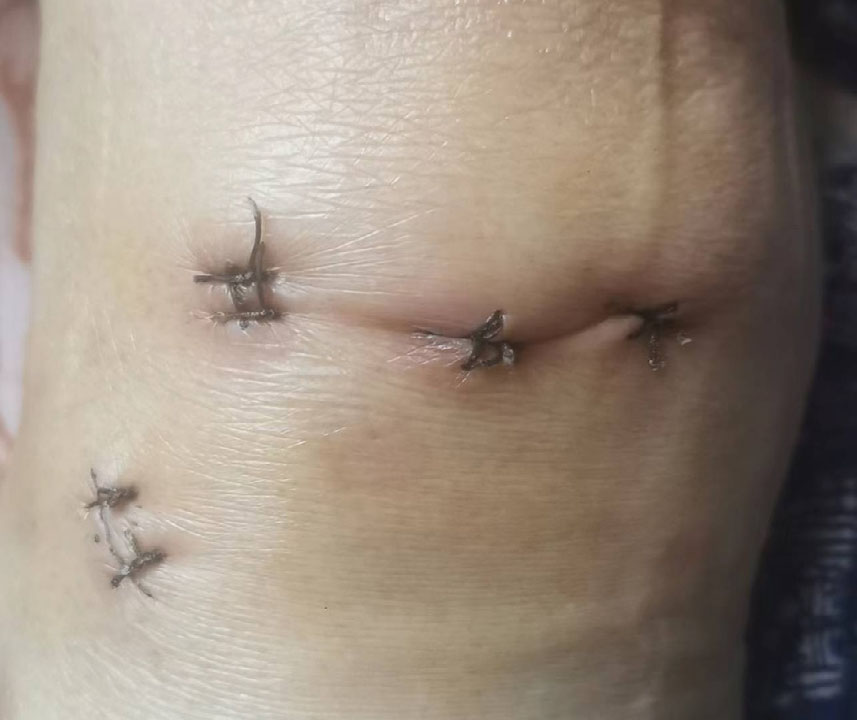
Wound image on the 12th postoperative day showed the incision had healed well.

The patient underwent epidural anesthesia complemented with intravenous sedative drugs and was placed in a supine position. The steps for the linear chain fixation method were as follows: (i) with the knee flexed at 40°, a standard anteromedial and anterolateral arthroscopic approach was employed. Under the arthroscope, the diagnosis of complete MMRPT (Figure 2A) and mild degeneration of osteoarthritis was confirmed. The MMRPT stump was then reconditioned, and the cartilage and cortical bone below it were removed with a curette to locate the inner opening of the tibial tunnel. (ii) A supplementary high‐positioned posteromedial knee entry was made; a cannulated needle was passed through the MMRPT stump (Figure 2B), and a single polydioxanone (PDS) suture and a single looped two‐strand #2 Arthrex non‐absorbable surgical suture were introduced. The two free ends of the suture passed through the suture loop of the folded end, forming a single‐strand double‐loop ligation fixation of the MMRPT stump (Figure 2C). (iii) A tibial tunnel (4.5 mm in diameter) was established using a locator. The inner opening was located as described previously, and the outer opening was located under Gerdy's tubercle. (iv) The middle folded part of a traction wire (PDS suture) was inserted into the joint cavity *via* the anteromedial entry below the patella. One of the two strands of the surgical suture that ligated the MMRPT stump was then removed from the tibial tunnel to the outside (Figure 2D). The end of the single‐strand surgical suture was passed through the loop part of the buckle‐strap titanium plate. Then, the single‐strand surgical suture was brought into the joint cavity via the tibial tunnel with the help of the folded traction wire, and exited from the anteromedial entry below the patella by the folded part of the PDS suture (Figure 2E). (v) After organizing the operating channel, the two surgical sutures were tied and pushed to the MMPR stump. The surgical suture that ligated the MMPR stump was then combined with the titanium plate loop of the locking strap in the joint cavity to form a mechanical traction complex (Figure 2F). (vi) The loop of the locking strap titanium plate had an adjustable length and was unaffected by its combination with the double‐stranded surgical suture. Its two free ends were tightened alternately and evenly via the locking mechanism to gradually move the micro titanium plate close to the bone at the outer opening of the tibial tunnel. (vii) After assessing the shape, tension and stability of the MMPR stump under arthroscopy (Figure 2G), once satisfied with the result, the two attached lines of the locking strap steel plate were tied and reinforced (Figure 3). The surgical video image has been provided in the Video S1.

### Postoperative Follow‐up

**FIGURE 4:**
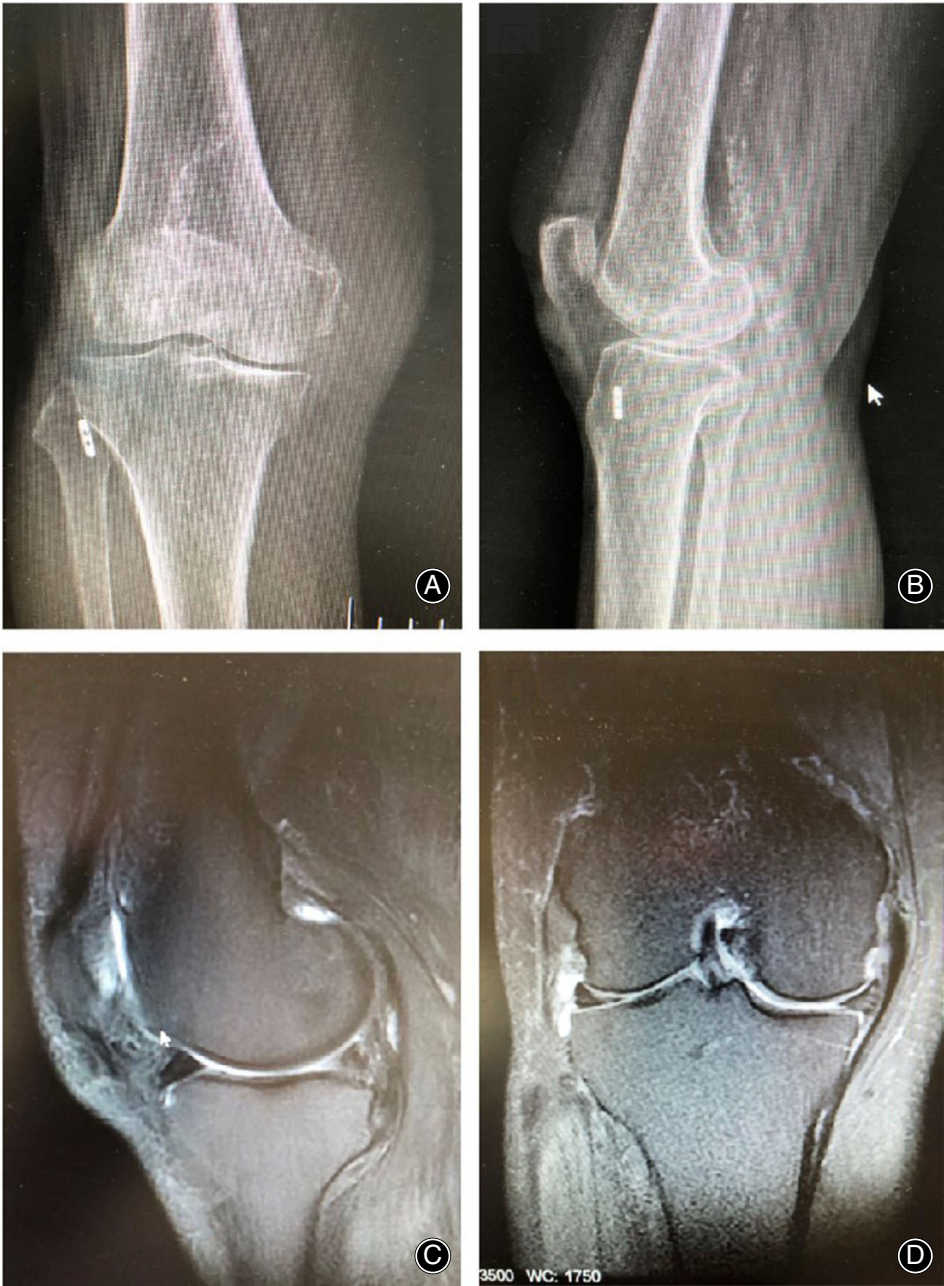
X‐rays and magnetic resonance imaging (MRI) of the affected knee joint at 7 months follow‐up. (A, B) X‐rays of the knee joint showed good positioning of the micro titanium plate at the external mouth of the tibial tunnel. (C, D) MRI showed that the deficit in the posterior root of the medial meniscus was filled and occupied by the medial meniscus stump reconstructed by the suture anchor (C), without an increase in protrusion deformity (D).

**FIGURE 5:**
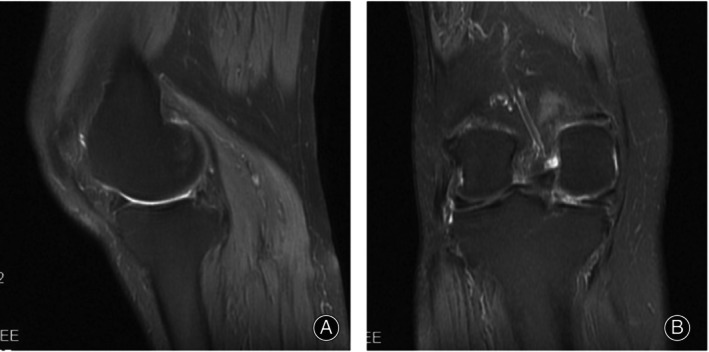
Magnetic resonance imaging (MRI) of the affected knee joint at 1 year follow‐up. (A) Sagittal MRI showed the presence of solid tissue at the posterior root attachment. (B) Coronal MRI showed that the medial meniscus was located between the medial tibiofemoral joint, with no obvious protrusion deformity.

On postoperative day 12, the incision had healed well (Figure 3), and the sutures were removed. At the 7‐month follow‐up, the patient was able to walk painlessly, although she exhibited quadriceps atrophy compared with the contralateral side; her muscle strength was at level 4, and squatting was limited. The Lysholm score was 87, and the VAS score was 1. X‐rays of the knee joint showed good positioning of the micro titanium plate at the external mouth of the tibial tunnel (Figure 4A,B). Magnetic resonance imaging showed that the deficit in the posterior root of the medial meniscus was filled and occupied by the medial meniscus stump reconstructed by the suture anchor (Figure 4C), without an increase in protrusion deformity(Figure 4D). At the 1‐year follow‐up, the patient experienced no pain during normal activities, with joint extension of 0° and flexion of 110°. The VAS score was 0, and the Lysholm score was 76. Sagittal MRI showed the presence of solid tissue at the posterior root attachment (Figure 5A). Coronal MRI showed that the medial meniscus was located between the medial tibiofemoral joint, with no obvious protrusion deformity (Figure 5B).

## Discussion

### Surgical Technique for MMPR Repair

MMPR tears are relatively common in clinical practice and may progress to spontaneous osteonecrosis of the knee and early osteoarthritis.^13^ As shown in Table 1,^14^, ^15^, ^16^, ^17^, ^18^, ^19^ the most commonly employed surgical technique currently involves using a linear construct to connect the MMRPT stump via an anteromedial approach beneath the patella, followed by the creation of a tibial tunnel through which the linear construct is pulled. It is then fixed in place using cortical bone screws, button plates, suture anchors or bone bridges.^20^ While this method is readily applicable and has achieved certain therapeutic effects, it typically presents two challenges.^21^, ^22^ First, the operational depth is substantial, and the space is narrow when connecting the MMRPT stump with a linear construct. The surgical instruments used, such as suturing hooks or suturing guns, are prone to damaging the articular cartilage surface of the medial femoral condyle. Second, after completing the knotting or anchor compression fixation of the end of the linear construct, the tension of the MMRPT stump often decreases compared with pre‐fixation, directly affecting its overall stability and postoperative outcomes.

**TABLE 1:** Cases of medial meniscus posterior root tear: Reported in the literature recently

Report	Age (year/sex)	Publication time	First symptoms	Complication	Path mechanism	Treatment	Reference
Qalib *et al*.	46/F	2022	Intermittent chronic left knee pain	Meniscal ossicle	No recent history of trauma, surgeries	Arthroscopic ossicle resection and meniscal root repair	Qalib *et al*.^14^
Hiranaka *et al*.	34/M	2019	Right knee pain	Bilateral anterior cruciate ligament rupture	History of trauma	Transtibial pullout repair and anterior cruciate ligament reconstruction	Hiranaka *et al*.^15^
Wasilewski *et al*.	51/F	2023	Acute onset right knee pain	Baker's cyst	No recent history of trauma, surgeries	Transtibial pullout repair	Wasilewski *et al*.^16^
Ohno *et al*.	78/M	2022	Right popliteal pain	A varus mechanical axis	No recent history of trauma	Medial meniscus posterior root reconstruction and high tibial osteotomy	Ohno *et al*.^17^
Okazaki *et al*.	67/F	2020	Right knee pain	‐	History of trauma	Transtibial pullout repair using a single tibial tunnel	Okazaki *et al*.^18^
Kubo *et al*.	75/F	2021	Right popliteal pain	Myasthenia gravis and hypertension	No recent history of trauma	Transtibial pull‐out repair	Kubo *et al*.^19^

### Potential Advantages of the New Linear Chain Fixation

The new linear chain fixation method is recommended during MMPR fixation, as it offers several potential advantages. First, the phenomenon of local trauma and relaxation caused by suture anchors can be avoided. In this method, the suture tied to the residual part of the medial meniscus and the loop part of the buckle‐strap titanium plate are combined into a linear chain mechanical complex, and the tension of the posterior root stump is gradually increased by pulling on the two attachment lines at the external mouth of the tibial tunnel to achieve a satisfactory degree. Second, the length of the linear structure of the fixed stump is not longer, than the full length of the tibial tunnel, which effectively controls mechanical instability caused by the elastic deformation of linear materials. Additionally, the two attachment wires fixed on the micro titanium plate prevent a deterioration in stability caused by the lengthening of the loop.

### Surgical Skills of the New Linear Chain Fixation

The linear chain mechanical complex is a key step in the surgical procedure. First, one of the two surgical stitches is returned to the joint after the loop ring is passed, which can be achieved via the PDS line or directly with slip pliers. After the loop ring is combined, the surgical stitches must be firmly tied and the thread pushed to the posterior root. In addition, when the two lines are gradually collected, do not push too hard; otherwise, it may damage the fixing part of the residual posterior root.

### Conclusion

In conclusion, this technique offers advantages, such as a simplified instrument configuration, maintenance of natural anatomical shape, reliable stump fixation.

## Authors' contributions

Chen J, Niu H and Li M collected the data. Dong Z, Gu J, Bai W and Chen B helped the date analysis and statistics. All authors took part in drafting the manuscript. All authors read and approved the final manuscript.

## Funding Information

This study is funded by Hebei Province key research and development plan project.

## Conflict of Interest Statement

The authors had no any personal, financial, commercial, or academic conflicts of interest.

## Ethics Statement and Consent to Participate

This study was conducted in accordance with the Declaration of Helsinki and approved by the ethics committee of Third Hospital of Hebei Medical University (K2023‐086‐1). Written informed consent was obtained from the participant.

## Supporting information


**Video S1.** Arthroscopic Linear chain fixation for the treatment of medial meniscus posterior Root Tear.

## Data Availability

All data generated or analyzed during this study are included in this published article.
